# Implementation and use of electronic synoptic cancer reporting: an explorative case study of six Norwegian pathology laboratories

**DOI:** 10.1186/s13012-014-0111-2

**Published:** 2014-08-20

**Authors:** Bettina Casati, Hans Kristian Haugland, Gunn Marit J Barstad, Roger Bjugn

**Affiliations:** Department of Pathology, Akershus University Hospital, Lørenskog, Norway; Department of Pathology, Haukeland University Hospital, Bergen, Norway; Department of Pathology, Stavanger University Hospital, Stavanger, Norway; Department of Research Administration and Biobanking, Oslo University Hospital, Kirkeveien 166, NO-0407 Oslo, Norway

**Keywords:** Checklist, Electronic health records, Information systems, Professional practice, Quality improvement

## Abstract

**Background:**

The information contained in histopathology reports on surgical resections of cancer is fundamental for both patient treatment and cancer registries. Electronic synoptic histopathology reporting is considered superior to traditional narrative reporting with respect to both completeness and feasibility of data use. An electronic template for colorectal cancer reporting was introduced in Norway in 2005, but implementation has varied greatly between different pathology departments. In 2012, four pathology departments and the Norwegian Cancer Registry started a new initiative on electronic cancer reporting. As part of this initiative, this study was undertaken to learn more about factors influencing implementation and use.

**Methods:**

Qualitative and quantitative data were obtained from six of the 17 public pathology departments in Norway using explorative case study methodology. Methods included document studies, semi-structured interviews with key informants, and audits on actual template use. A systematic analysis of data was conducted based on theoretical models for project management, stakeholder engagement, and individual acceptance of new information technology.

**Results:**

Most key informants had a positive view on synoptic reporting, and five departments had tested the electronic template. Of these, four had implemented the template while one department had decided not to implement it due to layout concerns. Of the four departments using the template in daily routine, one had compulsory use, two consensus based use, while the fourth had voluntary use. Annual average usage of the electronic template in the three departments with compulsory or consensus based use was 92% compared to 53% in the department with voluntary use.

**Conclusions:**

There was a general positive attitude towards electronic synoptic reporting. Reasons for not implementing the colorectal template were specific technical and quality issues not adequately addressed by the project organization having developed the template. A formal assessment of project outcomes with a task force handling such technical issues should accordingly have been established as part of the project. After an organizational decision on implementation, perceived job relevance and practical benefits are factors important for individual template use. Consistent high long-term usage was related to a departmental environment with a consensus based decision on use.

## Background

Surgical removal of the malignant tumor is in most instances the primary goal for treatment of patients with cancer. The resected cancer specimen is always analyzed in a pathology laboratory. The information provided in the histopathology report is important not only for further patient treatment, but also for having adequate cancer registries. The histopathology report has traditionally been in a free text (narrative) format. Many studies have however shown that the use of checklists or structured text elements improve the quality of the histopathology report with respect to the presence of key information deemed important for patient treatment [1-3]. With the introduction of electronic health records, structured histopathology reporting can be done using predefined drop down menus with automated coding. Because the information is arranged in discrete data fields, such electronic synoptic information can be automatically extracted and transmitted electronically to regional or national registries [3-5].

With these benefits in mind, the Norwegian Society of Pathology and the Cancer Registry of Norway collaborated on the development of electronic synoptic histopathology reporting on cancer from 2003 to 2006. The first template introduced was for colorectal carcinoma resections [4]. The template was designed using discrete data fields with predefined values, and it was compliant with all national information technology standards. Further, it was fully integrated into the two laboratory information systems being used by pathology laboratories in Norway at that time. Seven of the then 19 public pathology laboratories participated in the project. A national survey undertaken in 2008 showed that usage of the template varied greatly between pathology departments [6]. Some laboratories had a compliance rate above 90%, while others had not implemented the template at all. A study on long-term usage in one of the departments first having implemented the template, showed that usage was high (>90%) and consistent in the five-year period evaluated [1].

In 2012, four pathology departments and the Cancer Registry of Norway started a new initiative on structured electronic synoptic histopathology cancer reporting [7]. Given the previously observed great variation between pathology departments regarding the implementation and use of the template for colorectal cancer, it was decided to undertake a study among selected pathology departments to better understand factors affecting implementation and use.

## Methods

### Setting

Norway has a population of five million and healthcare is mainly public. The Directorate of Health is the public body responsible for establishing information and communication technology (ICT) standards in the healthcare sector. Since 2002, all hospitals are owned by one of four regional health authorities. In the period of 2003 to 2006, the regional health authorities established corresponding public ICT service provider organizations that operate ICT systems used by the hospitals. Prior to this, all hospitals were in charge of their own ICT services. As a general rule, no hospital can now acquire a new or substantially alter an existing ICT system without the prior approval of the regional ICT service provider and/or regional health authority.

### Methodology

Case study methodology is useful for studying contemporary events when relevant behaviors cannot be manipulated. Such methodology uses qualitative and quantitative data to try to explain why and how events happened, and what the results were. Theoretical models can be used for both designing such studies and for analyzing data. Exploratory case study methodology is used when the phenomenon in question is not fully understood, and when one wants to gather facts to form a hypothesis or to conduct further in-depth studies [8].

In this study, we used exploratory case study methodology to examine factors related to the implementation and use of structured electronic synoptic reporting in selected pathology departments. The three theoretical models used in the design of the study and for data analysis and interpretation are described below.

### Theoretical basis

#### Project management

**Figure 1:**
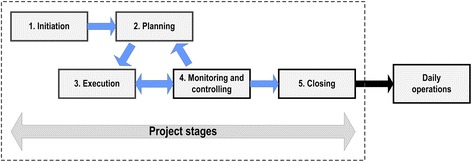
**From project to daily operations.** Illustration of a five-stage project model followed by daily operations.

Introduction of electronic synoptic reporting can be considered the deliberate change to technical and organizational subsystems that deal with information [9]. Such a change process covers initiation, development, implementation, operation, and maintenance of the new elements introduced [9,10]. In project management literature, this corresponds to a commonly used five-stage model of a project (initiation, planning, execution, monitoring and controlling, and closing) [11] followed by daily operations (Figure 1). This model was used to categorize and analyze information gathered from project documents, interviews with key informants, and the researchers’ own knowledge (see below).

#### Stakeholder model

**Figure 2:**
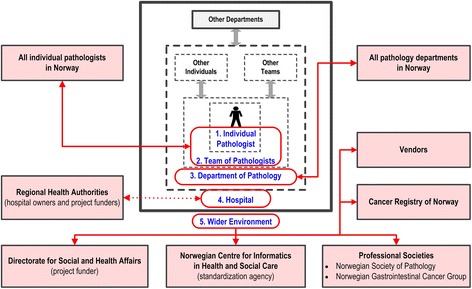
**Stakeholder model.** Five-level model of stakeholders in the healthcare system (modified after reference [12]). Stakeholders engaged in the previous project developing the electronic template for histopathology reporting of colorectal cancer is marked in red.

Effective implementation of new practices in medicine depends on a complex interaction between various stakeholders in the healthcare system, including patients, the professional, the healthcare team, the healthcare organization, and the wider environment [12]. In this study, we used a slightly modified stakeholder model for analyzing how different stakeholders may have been influenced by or have influenced, the implementation and use of structured electronic synoptic pathology reporting [13]. Such a multilevel stakeholder model is in line with the guidance document on project management published by the International Organization for Standardization [11]. The model includes the following five levels in the healthcare system (Figure 2):

1. The individual healthcare professional: In our study, the individual pathologists.
2. The team the individual healthcare professional works with: In our study, other pathologists as only pathologists undertake histopathology cancer reporting.
3. The department the individual healthcare professional is attached to: In our study, pathology departments.
4. The organization the individual healthcare professional is employed by: In our study, individual hospitals.
5. Individuals and organizations in the wider environment: In our study, dominantly organizations with a vested interest in pathology and/or related ICT services.

This model was used to categorize and analyze information gathered from project documents, interviews with key informants, and the researchers’ own knowledge (see below).

#### Technology acceptance model

**Figure 3:**
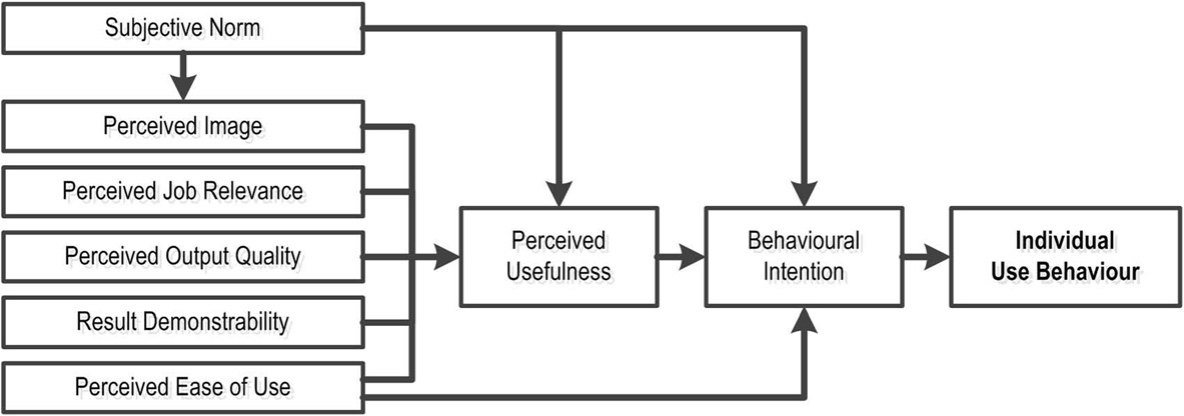
**Technology acceptance model.** Technology acceptance model illustrating factors affecting each individual healthcare professional’s adoption and use of new information technology solutions (modified after reference [15]). Definitions of terms are based on references [14,15]. Subjective Norm: An individual’s perception of how most people who are important to her think she should or should not use the IT system in question. The norm can either be perceived as voluntary or compulsory. Perceived Image: The degree to which an individual believes that using the IT system in question would enhance her status in her job related social system. Perceived Job Relevance: The degree to which an individual believes that using the IT system in question would be relevant to her job tasks. Perceived Output Quality: The degree to which an individual believes that using the IT system in question would improve the overall quality of her job tasks. Result Demonstrability: How apparent the benefits of using the IT system in question are to the individual. Perceived Ease of Use: The degree to which an individual believes that using the IT system in question would be free from effort. Perceived Usefulness: The degree to which an individual believes that using the IT system in question would enhance her job performance. Behavioral Intention: The degree to which an individual has formulated conscious plans to use (or not use) the IT system in question.

Successful long-term use is not guaranteed even after well-managed implementation of a new information system since healthcare professionals may not adhere to new guidelines and practices [12]. A theoretical ‘Technology acceptance model’ has been developed to try to untangle factors affecting each individual’s adoption and use of new information technologies [14,15]. In this study, a simplified version of this model (Figure 3) was used to analyze information gathered by interviews with key informants and the researchers’ own knowledge (see below).

### Sampling of cases

There are 17 public and two private pathology departments in Norway. Larger cancer resection specimens are mainly handled by public departments, and the study focus was therefore on these. To have a wide range of data and contexts, six departments (‘cases’) were to be included using predefined selection criteria (Table 1). Due to economic and time restraints, it was decided to limit the study to departments located in the Western and Southeastern health regions of Norway. The 12 public pathology departments located in these two regions handle approximately 75% of all histopathology samples processed annually by the 17 public departments in Norway [16]. One of the 12 departments was excluded from the study due to organizational issues because the department is the result of a recent merger between four previously independent university hospitals. Based on the selection criteria outlined in Table 1, six departments were contacted in the first instance. The remaining five served as a ‘reserve’ in case some of the six departments being approached did not want to participate.

**Table 1:** **Predefined selection criteria and characteristics of the six pathology departments participating in the study**

	**Department**					
**Selection criteria**	**1**	**2**	**3**	**4**	**5**	**6**
1. Previous Template Project	No	Yes	No	No	Yes	No
2. New Template Project	No	No	Yes	No	Yes	Yes
3. Univ./Non-Univ.	Non-Univ.	Univ.	Non-Univ.	Non-Univ.	Univ.	Univ.
4. No. of Consultants	6-10	>10	1-5	1-5	6-10	>10
5. Health Region	S-E	S-E	S-E	S-E	Western	Western
6. ICT System	Tieto	Siemens	Tieto	Siemens	Tieto	Siemens
**CRC Template Use**	No	Yes	No	Yes	Yes	Yes

**Previous Template Project**: Participation (n = 5) or non-participation (n = 12) in a previous national project on the development of an electronic template for histopathology reporting on colorectal cancer resections.

**New Template Project**: Participation (n = 4) or non-participation (n = 13) in a new project on further development of electronic synoptic cancer reporting.

**Univ./Non-Univ.**: University Hospital (n = 6) or Non-University Hospital (n = 11).

**No. of Consultants**: Size of department (defined as number of pathology consultants).

1 – 5 consultants (n = 8), 6 – 10 consultants (n = 6), more than 10 consultants (n = 3).

**Health Region**: Pathology laboratory located either in the South-Eastern (S-E) or Western Health Region.

**ICT System**: Using which one of the two laboratory information systems being used by all pathology laboratories in Norway (Doculive Pathology, Siemens, Norway or SymPathy, Tieto, Norway).

**CRC Template Use**: Using (Yes/No) the previously developed electronic template for histopathology reporting on colorectal cancer resections.

All six departments were approached by a formal letter explaining the background for the study, which type of information we requested, which key informants we would like to interview, and how the information gathered was to be used. A copy of the interview scheme was also submitted.

### Data sources and data collection procedures

The following four data sources were used:

1. Documents related to the previous development of the electronic template for colorectal cancer histopathology reporting.
  The study was given access to all previous project documents, from initiation to end report [4]. The documents were initially grouped and reviewed in accordance with the five project stages and the ensuing daily operations phase illustrated in Figure 1. Document drafts were not included in this review. A note summarizing the number of documents reviewed and the core content of the documents, related to the various stages, was prepared as part of this initial review. Project documents were also reviewed secondarily following information provided by key informants during the semi-structured interviews (see below) to extract more information on specific details.
2. Semi-structured interviews with key informants in the six pathology departments.
  Key informants were defined as head of department, chief medical advisor (if head of department was not a physician), quality advisor, and ICT advisor. The key informants accordingly included practicing pathologists. If the laboratory agreed to participate in the study, a semi-structured interview [17] with key informants at each department was undertaken by two board certified pathologists. Each interview lasted 1 to 1.5 hours and focused on general attitudes on electronic synoptic reporting, experiences with the previously developed structured electronic template for colorectal cancer (implementation and use), and views upon further development of structured electronic templates for cancer histopathology reporting. Notes were taken throughout the interview sessions and secondarily entered into a protocol corresponding to the semi-structured interview scheme used.
3. Archival departmental records on template use for colorectal cancer histopathology reporting.
  In cases where a pathology department had implemented the electronic template for routine colorectal cancer reporting, permission to extract anonymous data on actual template usage was requested. Using a combination of Systematized Nomenclature of Medicine codes for location (colon or rectum), carcinomas (World Health Organization classification system), and specimen type (resection), all colorectal specimens (both biopsies and resections) were identified in the pathology software systems for the relevant time periods. All specimens with less than three paraffin blocks (assumed not to be carcinoma resections) were discarded from the data file. The remaining histopathology reports were then manually screened in order to include only colorectal resections with primary carcinomas in the final data sets [1]. Whether or not the electronic template had been used for histopathology reporting was recorded. To investigate a possible change in template usage over time, data on usage were also linked to consecutive one-year periods after implementation.
4. The researchers’ personal knowledge of issues related to the development, implementation, and use of the electronic template (that is, the authors’ knowledge gained through firsthand experience or observation).
  All four researchers conducting this study have been involved in the development, implementation, and/or routine use of the electronic template for histopathology reporting of colorectal cancer. This background had provided the authors with knowledge gained through firsthand experience or observation on intra-departmental processes not being expressed in the written documents (data source 1) or semi-structured interviews (data source 2). Knowledge deemed relevant to the development of the template was added to the summary note prepared after review of the previous project documents (see above). Knowledge deemed relevant to the implementation and use of the template was added to the structured notes prepared shortly after the semi-structured interviews (see above).

### Data analysis

**Figure 4:**
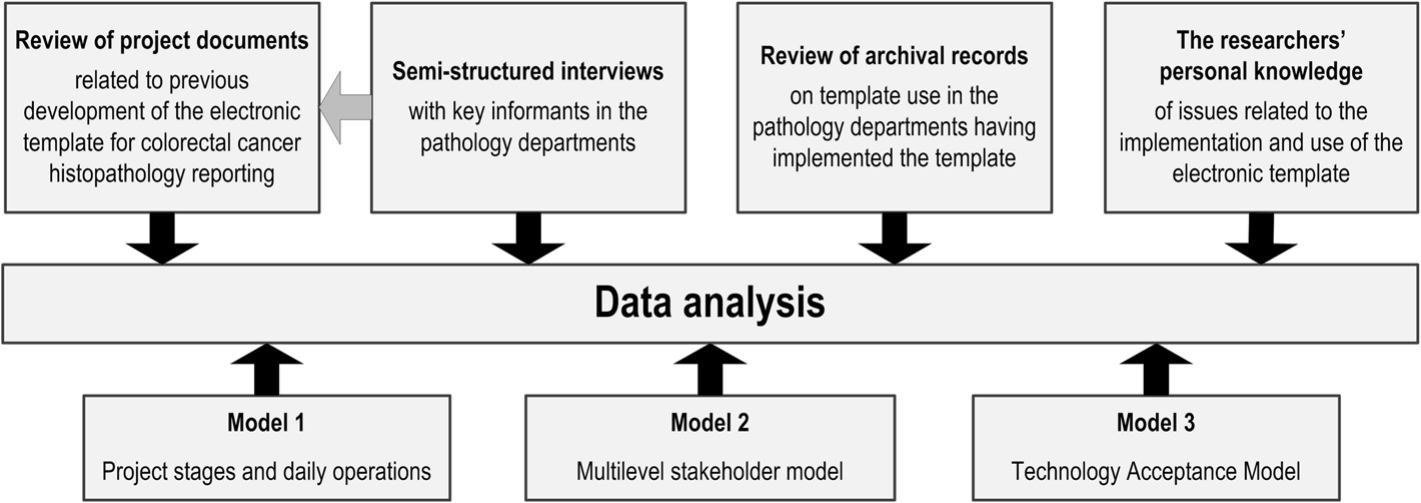
**Data analysis.** Illustration of how knowledge gained by the four various data sources were integrated and compared with the three theoretical models used.

Information obtained from review of the previous project documents (with supplementary researchers’ knowledge), interviews with key informants (with supplementary researchers’ knowledge), and archival departmental records on template use was initially collated and analyzed independently of each other. Information was then compared and analyzed to identify issues where additional information was needed (*e.g.*, secondary targeted review of project documents based on information obtained by semi-structured interviews). Following this, data collected were reviewed for common or contrasting information elements and compared with the three theoretical models used to evaluate the models’ appropriateness. Finally, collated information was analyzed in view of relevant literature to develop a better overall understanding of issues related to the implementation and use of the electronic template for histopathology cancer reporting (Figure 4).

### Ethics and consent

No medical research on human beings, human biological material, or personal health data was undertaken in this study. In accordance with the Norwegian act on medical and health research, no approval by a regional committee for medical and health research ethics was therefore required.

Although no departments are named, they may still be identifiable due to the specific selection criteria used (Table 1) and the limited number of departments present in the two health regions. Trying to fully anonymize departments (*e.g.*, by changing specific organizational characteristics) in this publication was deemed unrealistic [18]. However, all departments involved were informed about the intention to publish the results of the study, and we consider the information presented to be of little potential harm to the departments and individuals involved. With respect to disclosure of individuals, we have chosen not to present verbatim quotations [19].

## Results

### Documents related to the previous development of the electronic template for colorectal cancer histopathology reporting

Altogether 59 documents, ranging from one to 20 pages in length, were reviewed. The documents covered all project stages in the theoretical model illustrated in Figure 1, but none of the documents covered daily operations after formal project closure. Key information elements found in the project documents are given in Table 2.

**Table 2:** **Information on reviewed documents related to the previous development of the electronic template for colorectal cancer histopathology reporting**

**Stage**	**Number of documents**	**Key information elements**
Project initiation	6	Initiated as a response to various governmental requests for quality improvement in the healthcare sector.
Project planning	6	• Engagement of the Directorate of Health and the regional health authorities, which own Norwegian public hospitals, with respect to project aims and funding.
		• Targeted information about the project to all Norwegian pathology departments and individual pathologists.
		• Targeted engagement of national standardization agency and selected pathology departments.
		• *There were no written plans for how to facilitate implementation of the template in pathology departments. The underlying assumption was that this was not an issue as the template was fully integrated into the software systems already being used by all pathology departments in Norway.*
		• *Integration of pathology software systems (containing the template) with other software systems used by the hospitals was not addressed.*
		• *Specifications on graphical layout of template printouts were not addressed.*
Project execution	36	• Targeted engagement of pathology departments participating in the project, various medical societies/ interest groups, and vendors with respect to template development and other project related issues.
		• National standardization agency wrote protocol with guideline to be used by vendors in their software development of the template.
		• *Graphical layout of the template on printouts had been discussed in one project meeting where both vendors and participating departments were represented, but it had been decided that this issue could be addressed by the departments themselves as the software systems allowed local modifications.*
Project monitoring and controlling	9	Targeted use of three pathology departments in the testing, modification, and approval phase of the template developed by the vendors.
Project closing	2	Formal reports submitted to the Directorate of Health and the Board of the Norwegian Society of Pathology.
*Daily operations	0	There were no written plans for follow-up on project outcome (that is, successful implementation and use of the template) after formal project closing.

*This denotes issues related to maintenance, modifications and/or further development after a project is officially closed.

The documents are grouped according to the multistage project and daily operations model illustrated in Figure 1. Supplementary information details in *italic* address issues raised by key informants in semi-structured interviews thereby causing a secondary review of the original project documents.

The initial analysis showed wide engagement of stakeholders, including all individual Norwegian pathologists and all pathology departments (Figure 2). However, although all regional health authorities (owning the hospitals) were engaged, formal communication to the hospitals (as represented by the chief executive officer) engaged in the project was lacking.

With respect to implementation of the electronic template, the project strategy was to make the template an integral part of the two pathology software systems being used by all Norwegian pathology departments at that time, thereby avoiding any technical issues. Organizational decisions on template implementation for routine reporting were voluntary because the project organization had no formal power on the individual pathology departments.

With respect to the pathologists’ individual acceptance (and thereby use) of the electronic template, the project strategy was two-fold: to make the user interface as well-known as possible by integrating the template fully into the pathology software systems already being used, and to actively engage practicing pathologists in the development of the electronic template. Pathologists from all pathology departments participating in the project were accordingly engaged in the execution phase of the project, and pathologists from three of these departments were engaged in the monitoring and controlling stage.

When secondary review of the project documents were undertaken to investigate issues considered by key informants (see below) to hamper the implementation and use of the electronic template, written information on these specific issues were lacking in all instances. However, the researcher(s) personal knowledge added relevant supplementary information (see Table 2).

### Semi-structured interviews with key informants and archival departmental records on template use

All six pathology departments approached agreed to participate in the survey. Twelve key informants were interviewed, whereby six heads of departments, two medical advisors, two quality advisors, and two ICT advisors. Of the twelve key informants, eight were practicing pathologists.

#### General attitudes on electronic synoptic reporting

All key informants, with one exception, expressed a positive attitude on electronic synoptic histopathology reporting. This positivity was independent of whether the department had actually been using the colorectal template for routine histopathology reporting or not. Clinical relevance, useful reminder of parameters to report on, and ease of use were the most frequent commentaries.

Informants from two of the departments having implemented the template for routine reporting did however state that some of the practicing pathologists were strongly opposed to synoptic reporting, voicing concerns about losing the freedom of traditional narrative description.

#### Experience with synoptic reporting prior to the electronic template

One of the departments had a long tradition with synoptic histopathology cancer reporting using structured text elements in the histopathology report, while the other five departments had no experience with such structured reporting before the electronic template for colorectal cancer was developed.

#### Template testing and implementation

All six departments had decided to test the electronic template, but one department had not been able to do so due to technical issues related to integration with the hospital’s main electronic health record system. Of the five departments having tested the template, four had implemented it for routine reporting. These four departments had been using the electronic template for at least four years in daily routine. The one department deciding not to implement the template stated that they found the layout appearing on printouts to be graphically inadequate.

#### Template use

Of the four departments having used the template for routine histopathology reporting, one had compulsory use, two had a general consensus on use, and one had voluntary use. The department with compulsory use was the department with a long tradition of synoptic histopathology reporting (see above). Data on actual template usage in consecutive one-year periods are given in Table 3. The three departments with compulsory or consensus-based usage had an average annual use of 92% compared to 53% in the laboratory with voluntary use. The laboratory with voluntary use and one of the laboratories with consensus-based use were the two departments where informants had reported that some of the consultants had objected strongly to synoptic reporting (see above).

**Table 3:** **Actual usage of the electronic template for histopathology reporting on colorectal cancer resections in the four pathology departments having used the template in daily routine**

	**Department 1**		**Department 2**		**Department 3**		**Department 4**	
	**(Voluntary)**		**(Consensus)**		**(Consensus)**		**(Compulsory)**	
**Year-period**	**n**	**Use (%)**	**n**	**Use (%)**	**n**	**Use (%)**	**n**	**Use (%)**
1	118	37 (31.4)	251	214 (85.3)	225	210 (93.3)	146	136 (93.2)
2	127	80 (63.0)	266	238 (89.5)	242	222 (91.7)	132	127 (96.2)
3	137	81 (59.1)	302	276 (91.4)	233	203 (87.1)	156	153 (98.1)
4	123	64 (52.0)	232	208 (89.7)	237	223 (94.1)	149	144 (96.6)
5	128	71 (55.5)	-	-	249	231 (92.8)	186	186 (100.0)
	633*	**333 (52.6)**	**1,051**	**936 (89.1)**	**1,186**	**1,089 (91.8)**	**769**	**746 (97.0)**

*Total numbers and average use (%) are in bold.

None of the four departments using the template in daily routine had arranged any form of systematic educational sessions on template usage in connection with the implementation phase, and none had established a monitoring system on actual usage. The department with compulsory use had an underlying expectation that consultants signing out a histopathology report on colorectal cancer resection not using the template should state the reason for doing so, but there was no punitive action inherent in their administrative system or work culture if consultants deviated from the expected behavior.

#### Further development of electronic templates

Some informants expressed a wish for having the opportunity to add locally defined parameters in addition to the core elements being predefined in national templates.

With respect to engagement of stakeholders in possible further development of electronic templates, key informants from all six departments wanted the Norwegian Society of Pathology to take a lead with respect to defining relevant cancer parameters to be reported on. It was pointed out that the complex inter-organizational relationship between hospitals, regional ICT service providers, regional health authorities, and different public agencies made it very difficult to identify which organization should take the lead with respect to practical development and maintenance of electronic templates. A permanent and consistent organizational mechanism for keeping such templates up to date was considered essential for long-term success. Some informants also expressed a wish for electronic synoptic histopathology reporting to be part of the national cancer plan being developed by the Norwegian Directorate of Health.

#### Customer interaction and satisfaction

The department with a long tradition of compulsory synoptic histopathology reporting had a well-established practice whereby all drafts for new reporting templates where discussed with clinicians (oncologists and surgeons) before implementation. Although no formal customer satisfaction surveys had been undertaken, the department reported a positive attitude among clinicians for synoptic reporting, both in the form of structured text elements within a traditional narrative report and in the form of structured electronic data elements.

One of the departments with a general consensus on template use had undertaken a customer satisfaction survey [20] before implementing the electronic template for colorectal cancer. Several of the clinicians had then requested the use of synoptic reporting, generally based on personal experience from other hospitals where synoptic histopathology reporting had been introduced.

None of the key informants from the four departments having implemented the template for routine histopathology reporting reported negative feedback from clinicians.

## Conclusions

The aim of this exploratory case study was to gain better insight into factors affecting implementation and use of a structured electronic template for histopathology reporting of colorectal cancer. The discussion is focused on these two issues followed by methodological considerations, including an assessment of the three theoretical models used.

### Template implementation

#### General attitudes on electronic synoptic reporting

Information from the interviews showed that the large majority of key informants in the six pathology departments had a positive attitude towards electronic synoptic reporting, and that all six departments had actively investigated the possibility to implement the electronic template for routine histopathology reporting of colorectal cancer. Not implementing the template had quite specific causes.

#### Technical issues as a hindrance to implementation and use

Of the two departments not using the electronic template, one reported failure of implementation due to technical problems related to integration between the dedicated pathology laboratory information system and the hospital’s overall electronic health record. Review of documents related to the original development of the electronic template [4] showed that participating hospitals had not been actively engaged on an overall organizational level (Figure 2), and that integration between pathology information systems and other hospital ICT systems had not been considered (Table 2). In hindsight, formal engagement of the hospitals in the planning stage should have been done. This might have led to the identification, and thereby better management, of this issue. The stakeholder model illustrated in Figure 2 could also have been used for a systematic analysis of interactions between dedicated pathology software systems and other software systems being used in hospitals. In addition, a formal assessment of project outcomes should have been undertaken before the project was closed [21], and a dedicated task force for handling such technical issues should have been established as part of the project. Such a technical task force was part of a synoptic pathology reporting project in Ontario, Canada, and this facilitated implementation in their setting [5].

The other department not using the template reported successful implementation, but stated that they found the graphical layout of the template to be inadequate on printouts. Layout of pathology reports is important for proper communication [22]. Review of the original project documents showed that this issue had not been considered when the template was developed (Table 2). Layout on printouts had however been discussed in one end-user meeting during the execution stage of the project, but the consensus agreement among meeting participants was that this could be handled by local adjustments of the individual pathology software systems. Again, a dedicated task force for handling such technical issues might have solved this problem for the department in question. To our knowledge, no project on synoptic reporting has reported on how they have defined ‘quality’ with respect to graphical layout, and how they have ensured that this predefined standard was achieved throughout the communication chain between pathologists and clinicians. This issue should in our opinion be better addressed in future projects on synoptic histopathology reporting.

#### Further development of electronic template reporting

As pointed out by some of the respondents, the complex inter-organizational relationship between pathology laboratories, regional ICT service providers, regional health authorities, and ICT system vendors makes it crucial to establish a clear organizational mechanism for future development, implementation, and maintenance of electronic templates for histopathology cancer reporting. Similar organizational issues have also been raised by other studies on synoptic reporting [23,24]. We believe part of the success with synoptic reporting in Ontario, Canada is due to Cancer Care Ontario’s central organizational role, and that the relationship between participating organizations is formalized [5].

### Template usage

Actual use of synoptic reporting varied greatly in the four laboratories having implemented and used the electronic template for routine histopathology reporting (Table 3). Using the Technology acceptance model outlined in Figure 1, we will in the following discuss factors that may have influenced individual behavior with respect to template usage.

#### Subjective norm

##### **Compulsory or voluntary use**

Of the four departments having used the electronic template for routine reporting of colorectal cancer resections, one had compulsory use, two had a consensus-based decision on use, and the fourth had voluntary use. Although the department with compulsory template usage had a somewhat higher average user rate than the two departments with consensus-based usage (97.0% versus 91.8% and 89.1%), the absolute percentage number is just 5% to 8% higher. We believe this department’s long tradition of synoptic reporting (as structured text elements in the histopathology report) is more important than the formal departmental decision on compulsory usage. As there was no monitoring system for template usage or punitive measures if consultants deviated from expected behavior, we believe that a compulsory subjective norm mattered little with respect to template usage in our setting. A compulsory subjective norm may however influence significantly on individual behavior in settings with predefined organizational targets and ongoing monitoring of actual department and individual performance, such as in Ontario, Canada [25].

The department with voluntary use had a much lower template usage than the three departments with consensus-based or compulsory use. However, the department with voluntary use had few consultants (<6), and key informants reported that some of these opposed strongly to synoptic reporting. We believe the observed lower template usage in this department reflects the impact a limited number of individuals can have in a small organization, and not the effect of an organizational decision on voluntary or compulsory use.

##### **Opinion leaders**

With respect to a voluntary subjective norm, one of the two departments with a general consensus on voluntary template use had opinion leaders who were clearly in favor of synoptic reporting while the other had opinion leaders openly against. Despite this difference, both departments had a similar stable average template use around 90% (Table 3). Opinion leaders may have a significant impact on organizational change by influencing the behavior of co-workers [26], and the role of such individuals with a positive attitude on the intended change (‘champions’) has been highlighted in several studies on the implementation of electronic synoptic reporting [23,24,27]. While not downplaying such a factor, we propose that the ‘silent’ majority may adopt a new system independent of opinion leaders if the reasons for doing so are compelling for the individual health professional.

#### Perceived image

In our study, no department had established a monitoring system of template usage. Although one cannot exclude the possibility that individual pathologists monitor their own, and possibly others, reporting practices, our findings do not support such self-perceived image enhancement to be a significant factor for template use.

#### Other elements in the technology acceptance model

With respect to other elements in the technology acceptance model (perceived job relevance, perceived output quality, result demonstrability, and perceived ease of use; see Figure 3), our data do not allow further analysis. However, in other studies on electronic template reporting in Norway, time-saving benefits (‘result demonstrability’) [4] and ease of use [1] were judged to be contributing factors for using electronic template reporting in daily routine.

### Methodological considerations

#### Assessment of the theoretical models used

##### **Project management model**

The model illustrated in Figure 1 is based upon a model commonly used in project management [11], and we found it relevant and useful for both initial and secondary document reviews. The model was also useful for organizing and analyzing verbal information provided by key informants.

##### **Multilevel stakeholder model**

We believe the generic stakeholder model used [12] is valid for the organizational context in which the present study was undertaken, and we found it relevant and useful for designing the interview scheme prepared for the semi-structured interviews with key informants. The model was also relevant and useful for organizing and analyzing information obtained from project document and interviews with key informants (Figure 2). In addition, we also believe the model can be useful for exploring interactions between different software systems used in the healthcare sector.

In a study on the implementation and use of a web-based synoptic reporting tool for cancer surgery in Nova Scotia, Canada, Urquhart *et al.* pointed out that a good understanding of a complex organizational environment is crucial for project success [24]. However, as Urquhart *et al.* also found in their study, successful implementation does not guarantee successful usage of a new electronic reporting tool. In the end, it is the individual healthcare professional’s adaption of the tool that determines success or not.

##### **Technology acceptance model**

In our study, we used a generic technology acceptance model depicting factors affecting individual healthcare professional’s adoption and use of new information technology solutions (Figure 3). This model was deemed relevant for exploring organizational differences with respect to template use (Table 3). However, the data gathered were insufficient to undertake a complete analysis of all elements in the model. In retrospect we believe additional in-depth interviews with pathologists not representing departmental leadership should have been undertaken to better understand the behavior of individual pathologists. Other theoretical models may accordingly be just as useful, or better, for explaining individual behavior [28,29].

#### ‘Insider-outsider’ research team

In 1981, Evered and Louis introduced the terms ‘inquiry from the outside’ and ‘inquiry from the inside’ to describe situations where the researcher is, respectively detached from the organizational setting under study or becoming an integral part [30]. Researchers being members of the population under study offer both advantages and disadvantages in qualitative research [31-33]. In our explorative case study, we considered personal experience on synoptic pathology reporting to be necessary for designing the study appropriately, to be accepted by key informants, and to fully understand information obtained. With just 200 practicing pathologists in Norway, no researcher being a pathologist can in reality be a complete outsider of the community of pathologists. Even if the definition of community is limited to an individual pathology department, being an outsider can be difficult as most pathologists will have been training or practicing in several departments. In our study, we considered this methodological issue to be most important for the interviews. We accordingly used a two-person pathologist team, where at least one of the two never had been affiliated with the department in question.

#### Uniqueness versus generalization

Case study research reports on singular ‘cases’. As such, the findings should not be considered to be ‘representative’ for an entire population. The reason for including six of 17 public pathology departments in Norway in this study was accordingly not to have a representative population, but to gather a wide variety of unique experiences on synoptic histopathology reporting from pathology departments. However, unique experiences can be the foundation for a discussion on general principles regarding organizational and individual factors affecting implementation and use of synoptic reporting. Although our study was explorative in nature, we believe our ‘explanatory’ approach with respect to the findings discussed above is both relevant and methodological valid.

## Summary

The majority of key informants had a positive view on electronic template reporting and four of six pathology departments had implemented the previously developed template for histopathology reporting of colorectal cancer for routine use. The two departments not doing so had specific technical or quality reasons for their decision. We found the theoretical models for project management and stakeholder engagement used in this study to be both relevant and useful for understanding factors affecting implementation and use of a new tool for histopathology reporting. An appropriate use of these models can facilitate further development of structured electronic reporting in the healthcare system.
